# A Novel Panel of 43 Insertion/Deletion Loci for Human Identifications of Forensic Degraded DNA Samples: Development and Validation

**DOI:** 10.3389/fgene.2021.610540

**Published:** 2021-03-11

**Authors:** Rui Jin, Wei Cui, Yating Fang, Xiaoye Jin, Hongdan Wang, Qiong Lan, Yuxin Guo, Chong Chen, Xingru Zhang, Bofeng Zhu

**Affiliations:** ^1^Key Laboratory of Shaanxi Province for Craniofacial Precision Medicine Research, College of Stomatology, Xi’an Jiaotong University, Xi’an, China; ^2^Clinical Research Center of Shaanxi Province for Dental and Maxillofacial Diseases, College of Stomatology, Xi’an Jiaotong University, Xi’an, China; ^3^Department of Radiology, The Second Affiliated Hospital of Xi’an Jiaotong University, Xi’an, China; ^4^Multi-Omics Innovative Research Center of Forensic Identification, Department of Forensic Genetics, School of Forensic Medicine, Southern Medical University, Guangzhou, China; ^5^College of Medicine and Forensics, Xi’an Jiaotong University Health Science Center, Xi’an, China; ^6^Medical Genetic Institute of Henan Province, Henan Provincial People’s Hospital, People’s Hospital of Zhengzhou University, Zhengzhou, China

**Keywords:** InDel, individual identification, developmental validation, degraded DNA, Chinese Hui group

## Abstract

Insertion/deletion polymorphism is a promising genetic marker in the forensic genetic fields, especially in the forensic application of degraded sample at crime scene. In this research, a novel five-dye multiplex amplification panel containing 43 highly polymorphic Insertion/deletion (InDel) loci and one Amelogenin gene locus is designed and constructed in-house for the individual identification in East Asian populations. The amplicon sizes of 43 InDel loci are less than 200 bp, which help to ensure that full allele profiles can be obtained from degraded DNA sample. A series of optimizations and developmental validations including optimization of PCR conditions, detection efficiency of the degraded and casework samples, sensitivity, reproducibility, precision, tolerance for inhibitors, species specificity and DNA mixtures are performed according to the Scientific Working Group on DNA Analysis Methods (SWGDAM) guideline. The results of the internal validation demonstrated that this novel InDel panel was a reliable, sensitive and accurate system with good tolerances to different inhibitors, and performed the considerable detection efficiency for the degraded or mixed samples, which could be used in the forensic applications.

## Introduction

Human identification (HID) is one of dominating tasks in the forensic genetic field. Polymerase chain reaction-short tandem repeat (PCR-STR) analyses on capillary electrophoresis platform, which have been regarded as the “gold standard” for HID for more than 20 years (Zhang et al., 2018), are not only applied in criminal justice cases, but also in other aspects which include the identifications of missing persons, paternity tests and mass disaster victims (Zietkiewicz et al., 2012; Li et al., 2017; Ambers et al., 2018). However, STR analysis sometimes may result in unreliable conclusions due to relatively large amplified fragments and high mutation rates, especially when the biological samples from crime scene investigation are degraded or rotten. Besides, STR have limitations due to the systematic error of stutter peaks during the PCR process particularly when analyzing the mixture samples (Bennett et al., 2019). These shortages to some extent limit the effective usage of the STRs in forensic genetics.

InDel is also a kind of length polymorphism that can be analyzed in the polymerase chain reaction – capillary electrophoresis (PCR-CE) platform. InDel loci distribute widely in the human genome. And millions of InDel loci have been identified since they were first discovered in 2006 (Mills et al., 2006; Genomes Project et al., 2015). InDel genetic marker performs the merits of both STR and single nucleotide polymorphism (SNP) such as wide distributions in human genome, low mutation rates and short amplified fragments, no stutter peaks and available for capillary electrophoresis (CE) platform (Bashir and Hassan, 2016). These merits make biallelic InDel markers to be widely used in forensic HID and biogeographic ancestor inference (Chen L. et al., 2019; Lan et al., 2019; Sun et al., 2019).

The Investigator^®^ DIPplex kit (Qiagen, Germany) that comprises of autosomal thirty InDel loci and an Amelogenin locus, is now commonly used for forensic HID in different populations (Tomas et al., 2016; Chen P. et al., 2019; Haidar et al., 2019; Liu et al., 2019). However, population genetic investigations based on this kit revealed that some InDel loci like HLD118 and HLD111 loci were low polymorphisms in Chinese different populations (Xie et al., 2018; Jian et al., 2019). In recent years, several multiple InDel panels have been developed for the individual identifications of Chinese different populations or forensic ancestry predictions (Pereira et al., 2009; Chen L. et al., 2019; Sun et al., 2019; Huang et al., 2020). We also previously constructed a HID panel containing 35 InDel loci with the InDel amplicons less than 350 bp, which showed high genetic polymorphisms in Chinese some groups like Hui and Mongolian ethnic minorities (Jin et al., 2019; Cui et al., 2020; Zhang et al., 2020).

DNA detection of the degraded samples posed challenges for the forensic geneticist. In PCR amplification of degraded DNA, longer amplicons were more susceptible for the amplification failure compared with small amplicons. STR loci with large amplicons were not suitable for the analysis of degraded DNA since the amplicon sizes of commercial STR kits (except miniSTR kits) used in forensic genetics were in the range of 100 to 450 bp (Takahashi et al., 1997; Alaeddini et al., 2010). InDel genetic markers could be amplified in short amplicons (usually less than 160 bp), and therefore which improved the amplification success for degraded DNA (Pereira and Gusmão, 2012). The present research is prone to select a set of novel InDel loci with smaller amplicon sizes (<200 bp) to meet the detection need of the degraded samples.

In the present research, a novel multiplex amplification panel containing one Amelogenin gene locus and 43 highly polymorphic InDel loci is self-developed for the human individual identification in East Asian populations. The amplicon sizes of 43 InDel loci are less than 200 bp, which helps to ensure that full allele profiles can be obtained from degraded DNA sample.

Before a novel panel can be applied to the routine laboratory workflow, it is essential to evaluate its performance. In this research, a series of developmental validations were performed to validate the system performance of this novel panel according to the validation guideline of SWGDAM (Scientific Working Group on DNA Analysis Methods, 2016).

## Materials and Methods

### Ethics Statement and Sample Collection

This research was conducted in accordance with the ethical principle for medical research involving human subjects recommended by the World Medical Association Declaration of Helsinki. Our research was permitted and overseen by the ethics committee of Xi’an Jiaotong University, Health Science Center (Approval Number: 2019-1039). Peripheral venous blood samples from 533 unrelated healthy Hui individuals living in northwest region of China were collected. Before the volunteer recruitment, we had plastered the posters which proclaimed our research purpose and basic requirement for the volunteers under the permission and assistance of local directors. We also set up an information desk in different residential communities to publicize our research and attract more people to participate in our research. People who noticed our posters and were willing to learn more about our research came to our sampling sites and proceed the following processes. All volunteers were asked to finish the questionnaire about their health states and then signed the written informed consents before the blood sample collections. All participants declared that they were Chinese Hui people whose families have lived in northwest regions of China for at least three generations. Every participant volunteered for this study and they could withdraw from our study. All data were kept strictly confidential and analyzed anonymously. Professional nurses helped us collect peripheral venous blood. Human tissue collected from autopsies with the consents of their family members and animal organs were used as input DNA in the tests of species specificity, tissues/organs concordances and degraded samples. Animal samples were acquired from experimental animal center of Xi’an Jiaotong University. Execution of experimental animals also followed the animal ethics guideline.

### Reference Samples, Loci Selections and Primer Designs

Population data of 43 InDel loci from Han Chinese in Beijing (CHB), Southern Han Chinese (CHS), Chinese Dai in Xishuangbanna (CDX), Kinh in Ho Chi Minh City, Vietnam (KHV), and Japanese in Tokyo (JPT) acquired from 1000 Genomes Project Phase 3 (Ensembl release 86) were used as reference population data set (Genomes Project et al., 2015). According to the criteria for InDel loci screening described in the reference (Jin et al., 2019), the InDel loci in this panel were selected from NCBI dbSNP database Build 150^1^ according to the revised criteria as follows: (1) Candidate InDel loci should be biallelic variations which located on the non-coding regions of autosomes; (2) The allelic frequencies of candidate InDel loci in reference population data sets should be between 0.4 and 0.6; (3) The insertion/deletion sequence length for each allele should be between 2 and 30 bp; (4) Pairwise InDel loci should comply to linkage equilibrium; (5) All candidate InDel loci should show no significant deviations from Hardy–Weinberg equilibrium in reference data sets.

Primers of the 43 InDel loci were selected to construct the multiplex amplification system in the end, which were designed by Primer 5.0 tool. The amplicon sizes of each InDel locus were less than 200 bp. Oligo software version 7.0 (Rychlik, 2007) was used to evaluate whether the secondary structures were existed among primer pairs. The amplification specificities of all the InDel primers were initially evaluated using the BLAST function of NCBI website^2^. InDel primers were labeled by four different fluorescent dyes (HEX, TAMRA, 6-FAM, and ROX), respectively. Org500 (Microread Genetics, Beijing, China) was labeled orange and used as internal size standard. InDel primers were synthesized by Microread Genetics, Beijing, China. Allelic ladders of 43 InDel loci this novel panel were constructed by the method of genetic engineering, which followed the method of the published research (Jin et al., 2019).

### PCR Amplification and Capillary Electrophoresis

The novel 43-InDel panel developed by ourselves were multiplex PCR amplification and genotyped on capillary electrophoresis platform. Unless otherwise stated, PCR reaction system contained 12 μl of Master mixture, 0.4 μl of Primer mixture, 1 μl of DNA template and 6.6 μl of nuclease-free water. PCR were performed on the GeneAmp PCR System 9700 Thermal Cycler (Thermo Fisher Scientific, South San Francisco, CA, United States) under the following conditions: 5 min of initial denaturation at 95°C, followed by 35 cycles of 45 s for 94°C, 60 s for 56°C, and 60 s for 72°C, and then the final extension at 60°C for 60 min. ABI 3500xL Genetic Analyzer (Thermo Fisher Scientific, South San Francisco, CA, United States) was used to separate the PCR products with POP-4 (Thermo Fisher Scientific, South San Francisco, CA, United States). Before the electrophoresis, each loading sample containing 8.5 μl of Hi-Di formamide, 1 μl of PCR product (or allelic ladder) and 0.5 μl of Size Standard Org500 were prepared together. The alleles were genotyped using the GeneMapper ID-X software v1.5 (Thermo Fisher Scientific, South San Francisco, CA, United States). Control DNA 9947A and 9948, and deionized water were used as positive DNA and negative control, respectively.

### Optimization of PCR Reaction

We established a variety of tests to find out the optimal PCR reaction components and thermal cycling parameters. For each test, 1 μl of control DNA 9948 was used as input DNA. The amounts of Master mixtures and Primer mixtures, annealing temperatures and cycle numbers were adjusted respectively (the tested parameter varied while other parameters were in accordance with the PCR conditions mentioned above). For the tests of different reaction volumes, each experimental volume was enlarged or reduced proportionally based on the 20 μl volume. The tests were performed at the following conditions:

Master mixtures: 9, 10, 11, 12, 13, 14, and 15 μl.

Primer mixtures: 0.1, 0.2, 0.3, 0.4, 0.5, 0.6, and 0.7 μl.

Annealing temperatures: 54, 55, 56, 57, 58, and 59°C.

Cycle numbers: 30, 31, 32, 33, 34, 35, and 36.

PCR volumes: 5, 10, 15, 20, and 25 μl.

### Developmental Validations of the Novel 43-InDel Panel

#### The Detection Efficiency for the Artificial Degraded and Casework Samples

A series of tests were performed to evaluate the efficiencies of this InDel panel for the detection of degraded samples. 10 ng of positive control DNA 9948 was digested by DNase I (1U) at 37°C for 0.5, 1, 1.5, 2, 2.5, 3, and 4 min, respectively. Artificial degraded samples were amplified by the novel 43-InDel panel and then the PCR products were genotyped by the CE platform. In addition to the artificially degraded samples, exfoliated cells acquired from fingernails, razor blade, toothbrush, and hair follicle were also analyzed by this InDel panel and Microreader 21ID STR kit (Microread Genetics, Beijing, China), and then the ratios of detected alleles were calculated.

#### Accuracy and Concordance Studies

Control DNA 9948 was amplified by the novel 43-InDel panel, and then the purified PCR products were sequenced by the Sanger method to evaluate the genotyping accuracies of amplified fragments. Sanger sequencing was completed by Sangon Biotech^®^ company (Sangon Biotech Co., Ltd., Shanghai, China). To ensure whether consistent genotyping results were acquired when DNA samples of different organs or tissues of the same person were amplified, tissues/organs concordance studies were performed by amplifying the different DNA samples extracted from lung, liver, bone, muscle, thyroid gland, hair follicle, brain, spleen, kidney, heart, blood, pancreas, and fingernail of the same individual.

#### Sensitivity, Inhibitor and Species Specificity Studies

A range of amounts of positive control DNA 9948 (5 ng, 2 ng, 1 ng, 0.5 ng, 0.25 ng, 0.125 ng, 0.0625 ng, 31.25 pg, 15.625 pg) were made to evaluate the sensitivity of this InDel panel. Each input DNA was genotyped three times. A series of concentration gradients of hematin (0, 100, 500, 1,000, 1,500, 2,000, 3,000, 4,000, and 5,000 μM) and humic acid (0, 50, 100, 150, 200, 250, 300, 400, and 500 ng/μl) were used to evaluate the anti-interference capability of this 43-InDel panel. 1 μl of inhibitors with different concentrations were added to the PCR system, respectively. The amount of positive control DNA 9948 was held constant at 1 ng in different assessments. Nine non-human DNA samples including duck, canine, pig, cattle, sheep, SD rat, Kunming mouse, horse, and pigeon were used to assess the species specificity of this panel. 1 ng of non-human DNA was amplified using 43-InDel panel according to the standard PCR conditions.

#### Reproducibility and Mixture Studies

Ten human blood samples were selected to evaluate the reproducibility of all the 43 InDel loci in this InDel panel. Each test sample was amplified for ten times based on this 43-InDel panel, and then the consistency of each genotyping result was compared. In another reproducibility study, two sets of positive control DNA 9948 were amplified by this panel and detected in two different laboratories independent each other (Microread Genetics, Beijing and Microread Genetics, Suzhou). In the mixture studies, mixed DNA were prepared in which positive control DNA 9947A and 9948 (original concentration: 5 ng/μl) were mixed with different ratios of 19:1, 9:1, 6:1, 4:1, 2:1, 1:1, 1:2, 1:4, 1:6, 1:9, and 1:19 (9947A: 9948).

#### Population Study and Statistical Analysis

An online tool–STRAF (Gouy and Zieger, 2017) was used to calculate the allelic frequencies, exact tests of Hardy-Weinberg equilibrium (HWE) at 43 InDel loci, and a series of forensic parameters including observed heterozygosity (Ho), expected heterozygosity (He), power of exclusion (PE), polymorphism information content (PIC), typical paternity index (TPI), match probability (MP), and power of discrimination (PD). Furthermore, we investigated the genetic relationships between Hui and the reference groups. A heatmap was conducted based on the insertion allelic frequencies of 43 InDel to visualize the genetic diversities of these loci on 27 groups. Pairwise fixation index (*F*_*ST*_) values between Chinese Hui and the reference groups were calculated using Genepop v4 software. Multidimensional scaling (MDS) was conducted based on pairwise *F*_*ST*_ values using *R* 3.4.4 software.

## Results and Discussion

### InDel Loci Selection, Primer Design and Fluorescence Labeling

**FIGURE 1:**
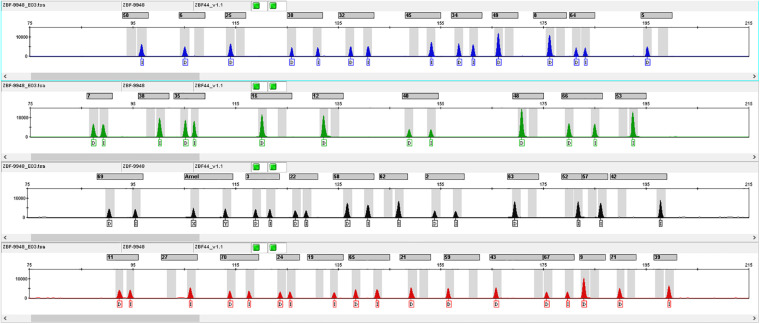
InDel profile of positive control DNA 9948. The number above the typing profile of the 43 InDel loci represented different InDel loci, which were shown in Supplementary Table 2.

According to the criteria for InDel selection, we finally chose 43 autosomal InDel loci to construct a multiplex amplification panel. The Amelogenin gene locus was added to the panel to identify the gender. The general information of 43 InDel loci was shown in Table 1. These 43 InDel loci distribute on all autosomes expect for chromosomes 2, 19 and sex chromosomes. The maximum insertion/deletion sequence length is 8 bp, which is found at rs5822909 and rs144537609 loci. The minimum and maximum amplicon sizes of 43 InDel loci are 87 and 199 bp, respectively, with a mean size of 147 bp. Profile of positive control DNA 9948 was shown in Figure 1.

**TABLE 1:** General information of selected novel 43 InDel loci.

Loci	Alleles	Chromosomes	Locations	Dyes
				
rs142159306	-/TTGAC	9	97445286:97445290	6-FAM
rs142281120	-/AG	9	112404922:112404923	6-FAM
rs146880183	-/TTC	14	37293145:37293147	6-FAM
rs3036240	-/CA	12	103890302:103890303	6-FAM
rs3092307	-/TTT	20	45379735:45379737	6-FAM
rs33990282	-/TAA	6	165777703:165777705	6-FAM
rs35700881	-/AAC	5	5051334:5051336	6-FAM
rs3830564	-/TGT	3	183762321:183762323	6-FAM
rs5852131	-/TC	3	117969173:117969174	6-FAM
rs6144473	-/AT	11	99625777:99625778	6-FAM
rs79287422	-/GA	4	113300378:113300379	6-FAM
rs10533337	-/TGAA	12	65437728:65437731	HEX
rs10537321	-/TG	1	222599319:222599320	HEX
rs10541072	-/TG	13	27598640:27598641	HEX
rs10589141	-/TAGAT	16	54019632:54019636	HEX
rs201844336	-/AG	10	116142130:116142131	HEX
rs3993057	-/AA	21	14110105:14110106	HEX
rs4019986	-/TG	1	201400512:201400513	HEX
rs5825145	-/TTTA	18	55685327:55685330	HEX
rs67941259	-/TG	1	107246975:107246976	HEX
rs10544053	-/CTTA	15	33489937:33489940	ROX
rs10555133	-/AA	1	160709188:160709189	ROX
rs10573809	-/AGTC	1	236547557:236547560	ROX
rs10584875	-/AA	6	61633814:61633815	ROX
rs10588341	-/GT	11	7255331:7255332	ROX
rs142392113	-/AAAGA	6	529405:529409	ROX
rs144537609	-/TCAGTTTG	22	20531188:20531195	ROX
rs147682692	-/GT	10	66286499:66286500	ROX
rs16646	-/TTTC	7	103760897:103760898	ROX
rs3043804	-/AG	12	80131192:80131193	ROX
rs3830885	-/AGG	6	47260269:47260271	ROX
rs5821525	-/TGAA	17	66434275:66434278	ROX
rs5892949	-/TA	8	85451803–85451804	ROX
rs10540867	-/AT	13	99305292:99305293	TAMRA
rs10555434	-/TTG	1	95796645:95796647	TAMRA
rs140025863	-/AG	3	79366895:79366896	TAMRA
rs142623177	-/TTTA	3	146076990:146076993	TAMRA
rs35974596	-/AAA	11	27379903:27379905	TAMRA
rs55714089	-/AG	10	99674013:99674014	TAMRA
rs5822909	-/AGAACACT	18	6573444:6573451	TAMRA
rs5882232	-/TAAAG	7	9775467:9775471	TAMRA
rs63064161	-/TCTT	12	2895679:2895682	TAMRA
rs63136060	-/AA	3	21875832:21875833	TAMRA

*These loci are selected from NCBI dbSNP database Build 150.*

These 43 InDel loci and one Amelogenin gene locus were divided into four groups according to the actual demand of the multiplex amplification. Primers of these InDel loci were labeled by fluorescent dyes with four different colors: the primers of rs142159306, rs142281120, rs146880183, rs3036240, rs3092307, rs33990282, rs35700881, rs3830564, rs5852131, rs6144473, and rs79287422 loci were labeled by the 6-FAM fluorescent dye (blue); the primers of rs10533337, rs10537321, rs10541072, rs10589141, rs201844336, rs3993057, rs4019986, rs5825145, and rs67941259 loci were marked with the HEX fluorescent dye (green); the primers of rs10544053, rs10555133, rs10573809, rs10584875, rs10588341, rs142392113, rs144537609, rs147682692, rs16646, rs3043804, rs3830885, rs5821525, and rs5892949 were marked by the ROX fluorescent dye (red); and the primers of rs10540867, rs10555434, rs140025863, rs142623177, rs35974596, rs55714089, rs5822909, rs5882232, rs63064161, rs63136060 loci and Amelogenin gene locus were labeled with the TAMRA fluorescent dye (yellow). The final concentrations of each primer ranged from 0.018 to 0.06 μM. Besides, the different fragments containing in the internal standard were labeled using Org500 (orange).

### Optimization of PCR Reaction System

Master mixtures – Master mixtures contained essential ingredients for the PCR reaction such as dNTPs, Taq DNA Polymerase, MgCl_2_ and buffer. The different amounts of the Master mixtures may influence the PCR efficiency. In this validation experiment, amplification efficiencies were tested with a series of amounts of Master mixtures, and the InDel profiles were shown in Supplementary Figure 1. Full profiles were acquired in all testing results of the different preset amounts of Master mixtures. With the increase of the amounts of Master mixtures (9–12 μl), the allelic peak heights increased correspondingly. When the input amounts of Master mixtures were 13 and 14 μl, no significant increase in allelic peak heights was observed. The highest peak height was observed when the input amount of Master mixtures was 15 μl, but imbalanced peak heights were also observed compared with other amounts. So we chose 12 μl as the optimal amount of Master mixtures.

Primer mixtures – Primer mixtures composed of the forward and reverse primers of 43 InDel loci and the Amelogenin gene locus. An appropriate amount (final concentration of each primer: 0.018 to 0.06 μM) of all the primers contributed to the high specificity and efficiency of multiplex PCR, so it was essential to study the optimal amount of the Primer mixtures in a novel panel during the process of panel construction. As shown in Supplementary Figure 2, when the input amount of Primer mixtures was 0.1 μl, lots of alleles dropped out in the fluorescent channels of TAMRA and HEX. Significant increases of allelic peak heights were observed when the input amounts of Primer mixtures increased from 0.2 to 0.4 μl. The allelic peak heights increased, but were not noticeable when the input amounts of Primer mixtures increased from 0.5 to 0.7 μl. Therefore, the optimal amount of Primer mixture was 0.4 μl.

Annealing temperatures – Annealing temperature is one of the important factors that affect PCR efficiency and product purity. Too high or low annealing temperature can lead to the increase of non-specific PCR products, resulting in lower PCR amplification efficiency (Rychlik et al., 1990). In this study, a series of annealing temperatures were set to find out the optimal annealing temperature. Complete DNA profiles were acquired at all testing results, but the better allelic peak height balances were observed when the annealing temperature was 56°C. So ultimately, we chose 56°C as the optimal annealing temperature.

Cycle numbers –In this study, 1 ng of template DNA was amplified at 30–36 cycles. In general, corresponding increases in allelic peak heights were observed with the increase of cycle numbers from 30 to 36 cycles. However, imbalance of peak heights was more obvious when the cycle numbers increased. In the routine practice, the cycle number of PCR was associate with the amount of template DNA and expected PCR product. A less cycle number may result in an insufficient amount of PCR product while PCR with too many cycle numbers produced abundant products, which may result in “fluorescent penetrant” phenomenon in the process of capillary electrophoresis. In this research, we chose 35 cycles as recommendatory cycle number when the template DNA was 1 ng.

Total volume of PCR – In the PCR volume experiment, 1 ng of control DNA 9948 was used as the template DNA. No amplification failure, allele dropout, or peak height imbalance were observed in the different reaction volumes of 5, 10, 15, 20, and 25 μl, respectively, which indicated that this panel was suitable for the different changes of reaction volumes. However, the small reaction volume was not recommended because it might result in small amounts of PCR product due to evaporation or nonstandard experimental process. So, we suggested the total reaction volume was at least 15 μl, an optimal reaction volume was recommended as 20 μl.

### Efficiency of the 43-InDel Panel for the Artificial Degraded and Casework Samples

**FIGURE 2:**
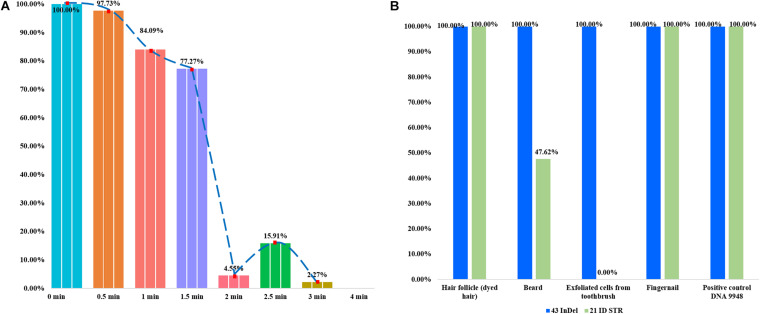
The detection ratios of 43 InDels in different degraded samples in triplicate. Each bar represented the number percentage of detected InDel loci. **(A)** The detection ratios of loci were presented when the control DNA 9948 was digested by DNase I (1U) at 37°C for 0.5, 1, 1.5, 2, 2.5, 3, and 4 min, respectively. **(B)** The detection ratios of loci were shown using the 43-InDel panel and Microreader 21ID STR kit for the casework sample.

In the forensic caseworks, biological samples from criminal scenes usually showed the different degrees of degradation because they were exposed to adverse environments such as light, heat, humidity or microorganism. It was important to develop a panel with high efficiency for the detection of degraded sample, which was also the purpose of the present research. Artificial degraded samples and casework samples were used to evaluate the efficiencies of this panel for detecting degraded samples. In Figure 2, each bar represented the percentage of the number of detected loci. As shown in Figure 2A, more than 70% InDel loci could be detected when the DNase I digest time was less than 1.5 min; only less than 20% loci could be detected when enzyme digest time was more than 3 min. In Figure 2B, genomic DNA extracted from four kinds of casework samples was genotyped using the 43-InDel panel and Microreader 21ID STR kit, respectively, and the ratios of the detected loci were calculated. For these four casework samples, all InDel loci could be successfully detected by this novel 43-InDel panel. Whereas only ten STR loci could be detected when the DNA of exfoliated cells acquired from razor blade was amplified by Microreader 21ID STR kit. No STR allele was detected when the DNA of the exfoliated cells acquired from toothbrush was amplified by Microreader 21ID STR kit. These results indicated that this panel performed well in the detection of degraded samples as well as casework samples compared with Microreader 21ID STR panel.

### The Tests of InDel Genotyping Accuracy and Tissues/Organs Concordance

To assess the InDel genotyping accuracy of amplified fragments, alleles of all InDel loci detected in control DNA 9948 were sequenced by the Sanger method. The InDel profiles acquired from the CE platform were consistent with the corresponding results of Sanger sequence, which showed that InDel profiles produced by this panel were accurate. In forensic caseworks, though blood sample was the most common biological material, other biological samples such as hair follicle or bone tissue were often examined as well so that it’s essential to evaluate the suitability of this panel for the detection of different tissues or organs. In the concordance study, genomic DNA templates extracted from 13 different organs or tissues from the same individual were amplified by 43-InDel panel, and then we compared the InDel profiles of different organs/tissues. As shown in Supplementary Figure 3, complete and identical profiles were acquired, which indicated that this panel could acquire the consistent profiles for different organs/tissues of the same individual.

### The Sensitivity, Inhibitor and Species Specificity Studies of 43-InDel Panel

**FIGURE 3:**
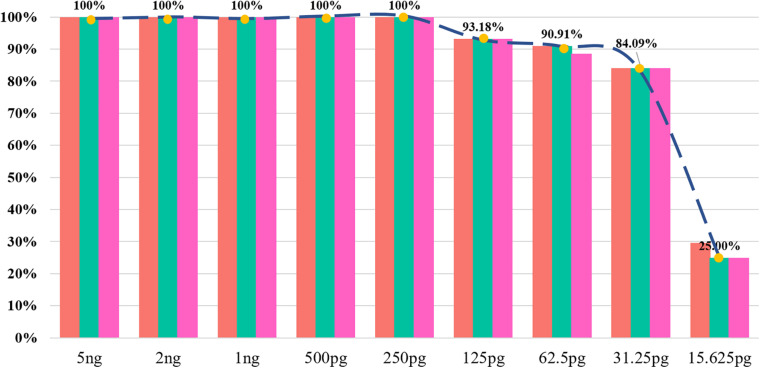
The detection ratios of 43 InDel loci in the sensitivity study in triplicate.

For forensic practitioners, it was not easy to acquire sufficient amounts of biological samples from crime scenes so that it’s essential for a panel to have the capacity for the detection of tiny DNA amount. The sensitivity study of this novel InDel panel was conducted by a range of DNA inputs from 15.625 pg to 5 ng. As shown in Figure 3, full profiles were obtained until the input DNA dropped below 250 pg. Allele dropout firstly occurred at rs5882232, rs10555434, and rs10537321 loci when the DNA input was 125 pg. Only 25% of the total alleles were observed when the DNA input was 15.625 pg. These results showed that the minimum input DNA amount that could acquire full InDel profile was 250 pg, so its sensitivity was 0.250 ng/20 μl, despite this, while 1 ng template DNA was recommended.

**FIGURE 4:**
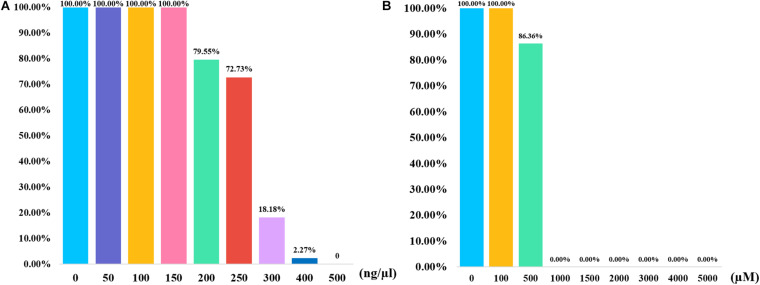
Bar plots summarizing the results of inhibition study for 43-InDel panel. **(A)** The results of the inhibition tests were obtained when various concentration gradients of humic acid were added to the PCR reaction. **(B)** The results of the inhibition study were shown when various concentration gradients of hematin were added to the PCR reaction.

In addition to the quality and quantity of the input DNA, the tolerance for the inhibitors also influenced the efficiencies of PCR amplification. In the stability study, the control DNA mixed with two different inhibitors were amplified and genotyped by this panel. Humic acid is a kind of component in soil, which may exist in the samples that are buried in soil. Researchers believed that humic acid may bind to DNA templates and result in low amount of available template, which could inhibit the PCR (Opel et al., 2010). As shown in Figure 4A, full profiles were obtained when the humic acid amounts were 50, 100, and 150 ng, respectively. Most of alleles dropped out when the humic acid amounts were 300 and 400 ng. No peak was detected when 500 ng of humic acid was added to the PCR system. Hematin is a kind of component in human blood, which may not be eliminated completely in the DNA extraction from blood samples. Hematin is a function of fluorescence quenching, which may result in a low PCR efficiency (Sidstedt et al., 2018). As shown in Figure 4B, full profiles were observed when 1 μl of 100 μM hematin was added to the PCR amplification. No allele was acquired when the concentrations of hematin ranged from 1,000 to 5,000 μM. The results revealed that this InDel panel performed good tolerance to different concentrations of two inhibitors.

To ensure that this novel panel showed no cross-reactivity between human and other species, BLAST tool was used to evaluate the species specificity of each primer sequence when we designed the InDel primers. In addition, nine non-human DNA samples were also used to evaluate the species specificity of this panel. The results showed that no peak was detected in these non-human DNA samples, which demonstrated this novel InDel panel performed no cross-reactivity between human and other species.

### Reproducibility and Mixture Studies

We performed two kinds of reproducibility tests to evaluate the reliability and accuracy of this panel. In the first test, consistent genotyping profiles were obtained for each sample and no allele was off the ladder bin. In the reproducibility evaluation by two different laboratories, coincident profiles were acquired for the same sample, which indicated that this novel panel performed the considerable reproducibility and could acquire reliable profiles in different laboratories.

It was necessary for a novel panel to have the ability for the genotyping of mixture DNA. In the mixture study (shown in Supplementary Figure 4), various artificial mixture samples were made with the known ratios (9947A and 9948 were mixed at 19:1, 9:1, 6:1, 4:1, 2:1, and 1:1), and the amount of male DNA remain unchanged (5 ng). Full alleles were detected at the ratios of 1:1, 2:1, 4:1, and 6:1. Peak heights of minor alleles decreased as the female DNA amounts increased from 5 to 30 ng. Peak heights of minor alleles dropped out at the ratio of 1:9. Thus, this novel panel could detect the mixture sample at the ratio of 6:1. The results revealed that it’s possible to acquire full profiles of the male DNA with this novel panel, even in the existence of an excess of female DNA.

### Population Genetics

Allelic frequencies of the novel 43 InDel loci in Hui and reference groups were shown in Supplementary Table 1. Forensic parameters of these 43 InDel loci were calculated based on the population data of 533 Hui volunteers, and the results were shown in Supplementary Table 2. No significant deviations from HWE were found in these 43 InDel loci after a Bonferroni correction (*p* = 0.05/43 = 0.0012). The insertion allelic frequencies of these 43 InDel loci in Chinese Hui group ranged from 0.3799 (rs55714089) to 0.5891 (rs144537609), with an average value of 0.4815. The He values ranged from 0.4716 (rs55714089) to 0.5005 (rs3993057 and rs10589141), with an average value of 0.4943, which indicated these 43 InDel loci had high genetic polymorphisms in Chinese Hui group. The mean values of MP, PD, PIC, PE, Ho, and He were 0.3802, 0.6198, 0.3719, 0.1852, 0.4965, and 0.4943 respectively. The combined PD and PE values were 0.999999999999999999147246 and 0.999852151, respectively, which indicated that these 43 InDel loci could be used for forensic individual identifications in Chinese Hui group.

**FIGURE 5:**
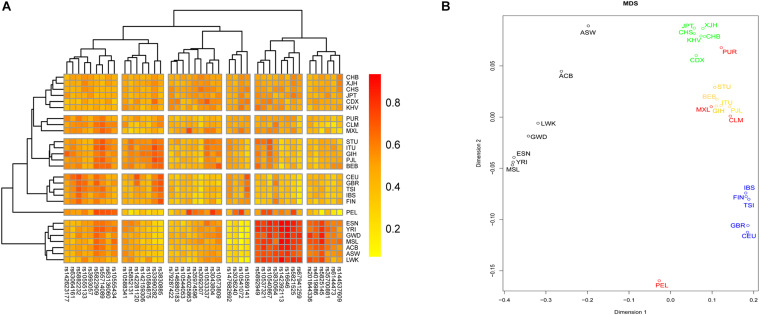
A heatmap and a multidimensional scaling (MDS) plot were conducted to evaluate the genetic relationships between Hui group and reference group. **(A)** A heatmap was conducted based on the insertion allelic frequencies of 43 InDel to visualize the genetic diversities of these loci. **(B)** A MDS plot was conducted based on pairwise *F*_*ST*_ values using R software.

We investigated the genetic relationships between Chinese Hui and reference groups based on these 43 InDel loci. A heatmap was conducted on the basis of the insertion allelic frequencies of 43 InDel loci to visualize the genetic diversities of these loci, and the result was shown in Figure 5A. For InDel loci, two main clades could be identified: fourteen loci with high insertion allelic frequencies in African groups clustered together; other loci were divided into four subclades according to the distribution characteristics of their insertion allelic frequencies. For 26 groups, all groups clustered into five major branches, roughly in line with their geographic location. The studied Hui group clustered with CHB firstly, and then clustered with East Asian groups, which indicated that Chinese Hui had close genetic relationships with East Asian groups.

A MDS plot was conducted based on pairwise *F*_*ST*_ values using *R* software, and the result was shown in Figure 5B. All groups clustered into four major branches: (1) seven groups from Africa clustered in the left part of the plot; (2) five groups from Europe clustered on the lower right side of the graph; (3) groups from South Asia clustered together and located on the right center of the plot; (4) groups from East Asia clustered together and on the top of the plot. The studied Hui group clustered closely with East Asia groups, indicating that there were close genetic affinities between Hui and East Asian groups.

We noticed that some novel InDel kits with high genetic polymorphisms in East Asian populations have been constructed such as AUCG 50 InDel, 32-plex InDel and 38-plex InDel kits. The AUCG 50 InDel kit contained 47 autosomal InDel loci labeled by five fluorescent dyes. However, amplified fragments of some loci in this kit were greater than 200 bp, which might result in allele dropouts when degraded samples were genotyped (Chen L. et al., 2019). For Pereira’s 38 InDel kit, the combined random matching probability (RMP) values of these 38 loci ranged from 10^–15^ to 10^–13^ in European different groups (Pereira et al., 2009; Manta et al., 2012). For this 43-InDel panel, we calculated the CPD values of 43 InDel loci in African, European, and East Asian groups using the population data acquired from 1000 Genomes Project Phase 3. The CPD value of 43 InDel in East Asian group was 0.9999999999999999770312, which was higher than the CPD values in African and European groups (0.9999999999999999198522 and 0.99999999999999999470465, respectively). The combined RMP value of the 43 InDel loci was lower than 10^–19^ in Chinese Hui group, indicating that these loci have higher cumulative discrimination power in Chinese groups compared with Pereira’s 38-plex InDel and Huang’s 32-plex InDel kit. The CPE value of these 43 InDel loci were 0.999887424, which indicated that this panel may be used as a promising tool in the paternity testing.

## Conclusions

In this study, a novel panel that could simultaneously amplify 43 InDel loci and one Amelogenin gene locus was developed. The maximum amplicon size of 43 InDel loci was 199 bp, which ensured that full allele profiles could be obtained from the degraded DNA samples. The results of the developmental validation demonstrated that this InDel panel was robust, sensitive and accurate, and performed the considerable efficiencies for the detection of the degraded or mixture samples. Population genetic evaluations in Chinese Hui group indicated that this 43-InDel panel was a powerful tool for the individual identification in Hui group.

## Data Availability Statement

The raw data supporting the conclusions of this article will be made available by the authors, without undue reservation.

## Ethics Statement

The studies involving human participants were reviewed and approved by the Ethics Committee of Xi’an Jiaotong University, Health Science Center and Southern Medical University (Approval Number: 2019-1039). The patients/participants provided their written informed consent to participate in this study. The animal study was reviewed and approved by Ethics Committee of Xi’an Jiaotong University, Health Science Center and Southern Medical University (Approval Number: 2019-1039).

## Author Contributions

BZ designed this multiple amplification system and was responsible for all the processes of this research. RJ and WC constructed and validated this multiple amplification system, analyzed the data, and wrote this manuscript. YF and XJ selected these 43 InDel loci. HW and YG assisted the experiments and data analyses. BZ, CC, QL, and XZ revised this manuscript. All authors have read and agreed to the published version of the manuscript.

## Conflict of Interest

The authors declare that the research was conducted in the absence of any commercial or financial relationships that could be construed as a potential conflict of interest.

## Footnotes

1 https://www.ncbi.nlm.nih.gov/snp/

2 https://blast.ncbi.nlm.nih.gov/Blast.cgi

## Supplementary Material

Supplementary Figure 1 InDel profiles for the optimization studies with a series of amounts of Master mixtures.

Supplementary Figure 2 InDel profiles for the optimization studies with different amounts of Primer mixtures.

Supplementary Figure 3 InDel profiles for the tissue/organ concordance studies using the different DNA extracted for 13 tissues or organs of the same individual.

Supplementary Figure 4 InDel profiles for the mixture DNA studies. Positive control DNA 9948 and 9947A were mixed with the different ratios of 19:1, 9:1, 6:1, 4:1, 2:1, 1:1, 1:2, 1:4, 1:6, 1:9, and 1:19, respectively.

Supplementary Table 1 Allelic frequencies of 43 InDel loci in 26 reference groups and Chinese Hui groups.

Supplementary Table 2 Forensic parameters of 43 InDel loci in Chinese Hui group (*n* = 533).
