# Supplementary figures and images for: A Novel Panel of 43 Insertion/Deletion Loci for Human Identifications of Forensic Degraded DNA Samples: Development and Validation
Source: Front Genet. 2021 Mar 11;12:610540. doi: 10.3389/fgene.2021.610540 (PMC7990895; doi:10.3389/fgene.2021.610540)

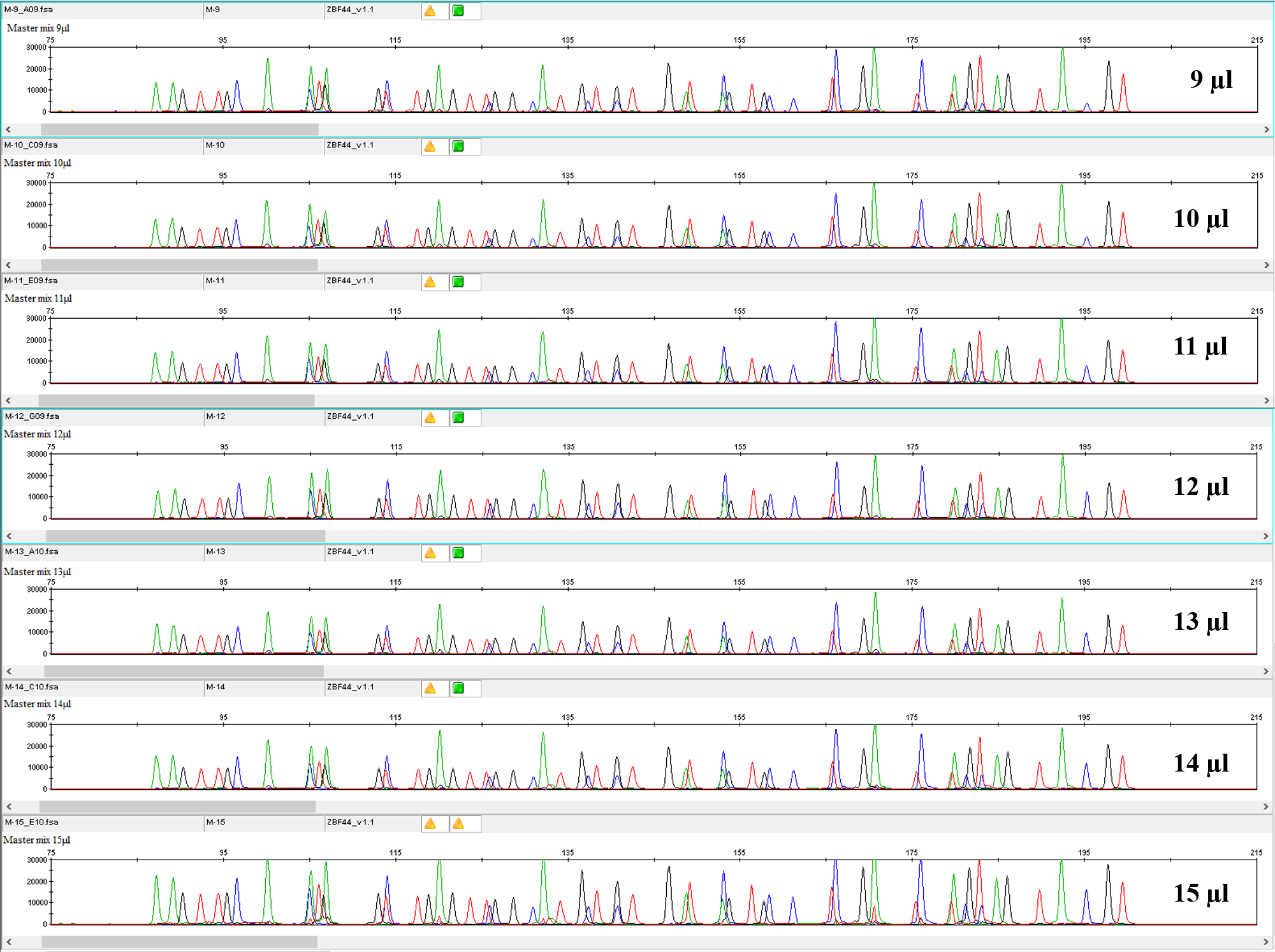

Supplement: Supplementary Figure 1 — InDel profiles for the optimization studies with a series of amounts of Master mixtures. [file Image_1.TIF]

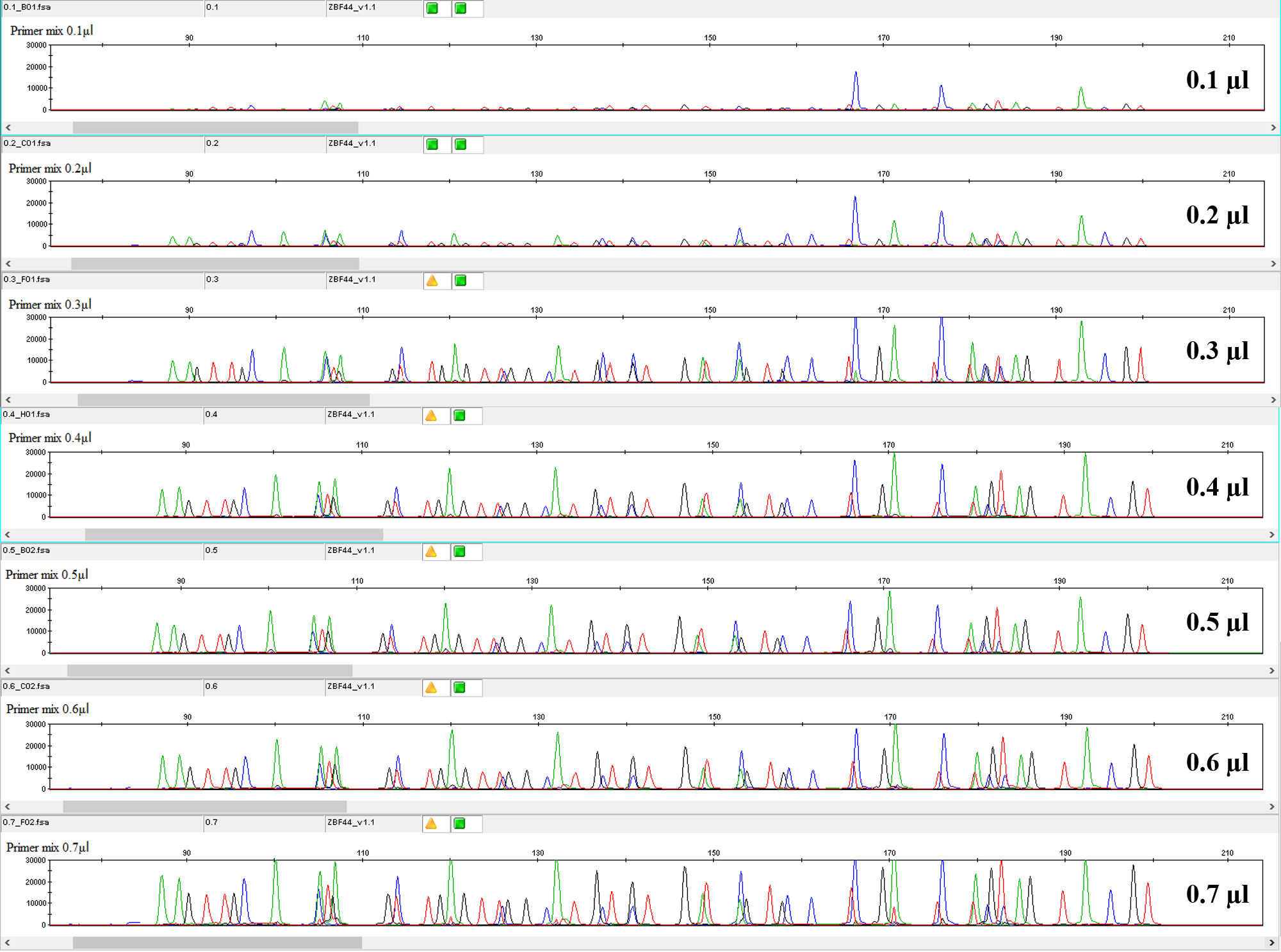

Supplement: Supplementary Figure 2 — InDel profiles for the optimization studies with different amounts of Primer mixtures. [file Image_2.TIF]

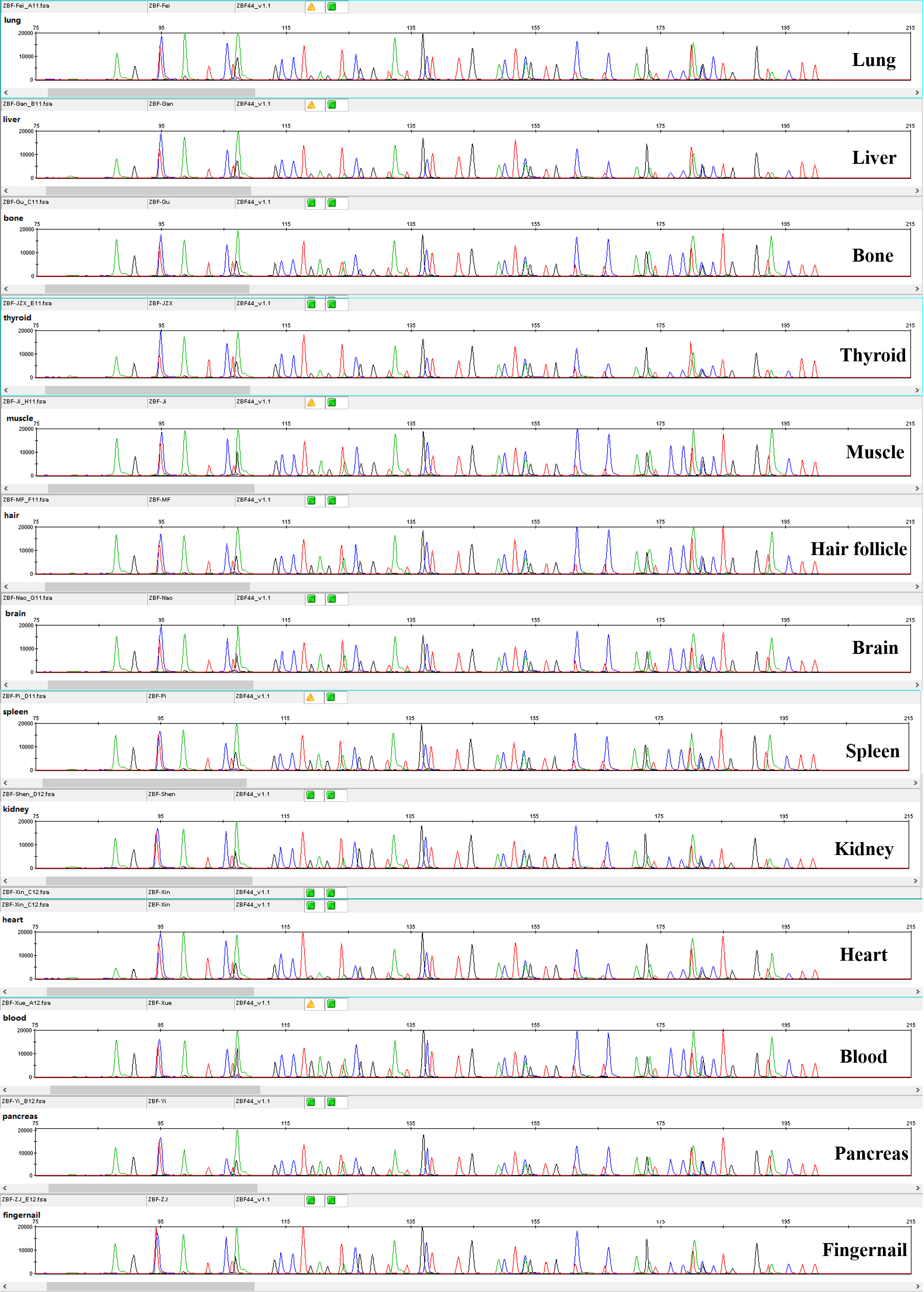

Supplement: Supplementary Figure 3 — InDel profiles for the tissue/organ concordance studies using the different DNA extracted for 13 tissues or organs of the same individual. [file Image_3.TIF]

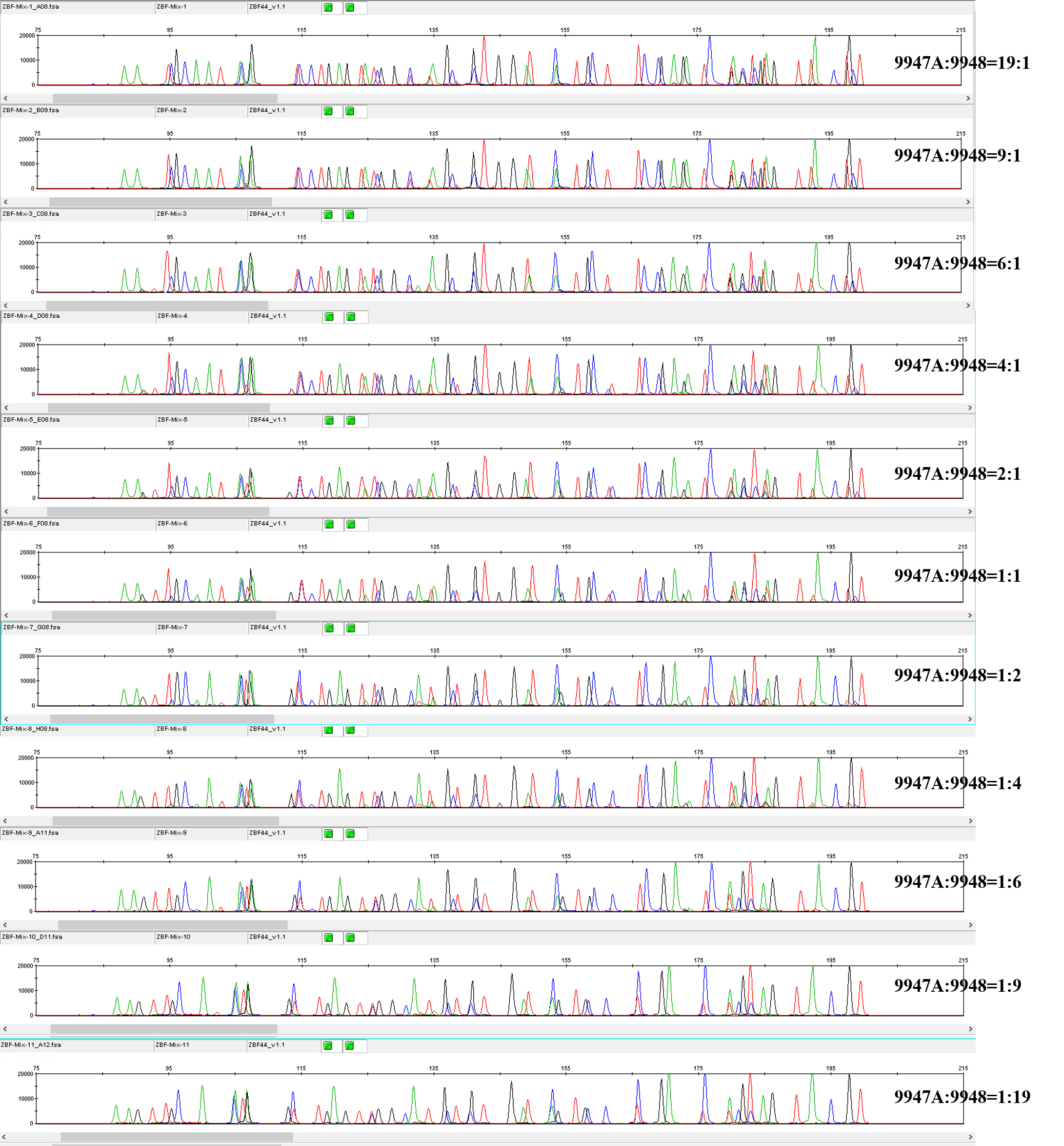

Supplement: Supplementary Figure 4 — InDel profiles for the mixture DNA studies. Positive control DNA 9948 and 9947A were mixed with the different ratios of 19:1, 9:1, 6:1, 4:1, 2:1, 1:1, 1:2, 1:4, 1:6, 1:9, and 1:19, respectively. [file Image_4.TIF]
