# Supplementary material for: A Novel Panel of 43 Insertion/Deletion Loci for Human Identifications of Forensic Degraded DNA Samples: Development and Validation
Source: Front Genet. 2021 Mar 11;12:610540. doi: 10.3389/fgene.2021.610540 (PMC7990895; doi:10.3389/fgene.2021.610540)
Supplement: Supplementary Table 2 — Forensic parameters of 43 InDel loci in Chinese Hui group (n = 533). [file Table_2.docx]

Supplementary Table 2. Forensic parameters of 43 InDel loci in Chinese Hui group (n=533).

| No | Loci | Insertion Frequency | He | PIC | MP | PD | Ho | PE | TPI | *p*HW |
| --- | --- | --- | --- | --- | --- | --- | --- | --- | --- | --- |
| 2 | rs63064161 | 0.4756 | 0.4993 | 0.3744 | 0.3712 | 0.6288 | 0.4897 | 0.1786 | 0.9798 | 0.648 |
| 3 | rs10555434 | 0.4522 | 0.4959 | 0.3727 | 0.3791 | 0.6209 | 0.4991 | 0.1867 | 0.9981 | 0.923 |
| 5 | rs33990282 | 0.5675 | 0.4913 | 0.3704 | 0.3988 | 0.6012 | 0.5272 | 0.2124 | 1.0575 | 0.086 |
| 6 | rs146880183 | 0.3996 | 0.4803 | 0.3647 | 0.3876 | 0.6124 | 0.4841 | 0.1739 | 0.9691 | 0.927 |
| 7 | rs10537321 | 0.47 | 0.4987 | 0.3741 | 0.3718 | 0.6282 | 0.4897 | 0.1786 | 0.9798 | 0.721 |
| 8 | rs5852131 | 0.3968 | 0.4792 | 0.3641 | 0.3862 | 0.6138 | 0.4784 | 0.1693 | 0.9586 | 1 |
| 9 | rs10584875 | 0.5122 | 0.5002 | 0.3749 | 0.3677 | 0.6323 | 0.4841 | 0.1739 | 0.9691 | 0.508 |
| 11 | rs10588341 | 0.4859 | 0.5001 | 0.3748 | 0.3749 | 0.6251 | 0.4991 | 0.1867 | 0.9981 | 1 |
| 12 | rs10541072 | 0.5394 | 0.4974 | 0.3734 | 0.3749 | 0.6251 | 0.4934 | 0.1818 | 0.987 | 0.869 |
| 16 | rs5825145 | 0.4493 | 0.4953 | 0.3724 | 0.3769 | 0.6231 | 0.4934 | 0.1818 | 0.987 | 0.934 |
| 19 | rs3830885 | 0.4869 | 0.5001 | 0.3748 | 0.4039 | 0.5961 | 0.5497 | 0.2348 | 1.1104 | 0.024 |
| 21 | rs10555133 | 0.5835 | 0.4865 | 0.3679 | 0.3719 | 0.6281 | 0.4615 | 0.156 | 0.9286 | 0.277 |
| 22 | rs63136060 | 0.5122 | 0.5002 | 0.3749 | 0.3889 | 0.6111 | 0.5253 | 0.2106 | 1.0534 | 0.262 |
| 24 | rs147682692 | 0.4869 | 0.5001 | 0.3748 | 0.3721 | 0.6279 | 0.4934 | 0.1818 | 0.987 | 0.784 |
| 25 | rs3036240 | 0.3968 | 0.4792 | 0.3641 | 0.38 | 0.62 | 0.4634 | 0.1574 | 0.9318 | 0.463 |
| 27 | rs10573809 | 0.4944 | 0.5004 | 0.375 | 0.3755 | 0.6245 | 0.5009 | 0.1883 | 1.0019 | 1 |
| 30 | rs142159306 | 0.4803 | 0.4997 | 0.3746 | 0.3633 | 0.6367 | 0.4728 | 0.1648 | 0.9484 | 0.249 |
| 32 | rs35700881 | 0.4906 | 0.5003 | 0.3749 | 0.3738 | 0.6262 | 0.4972 | 0.185 | 0.9944 | 0.936 |
| 34 | rs3092307 | 0.5366 | 0.4978 | 0.3737 | 0.398 | 0.602 | 0.5366 | 0.2216 | 1.0789 | 0.084 |
| 35 | rs4019986 | 0.4371 | 0.4926 | 0.371 | 0.377 | 0.623 | 0.4878 | 0.1771 | 0.9762 | 0.871 |
| 38 | rs201844336 | 0.4015 | 0.481 | 0.3651 | 0.3978 | 0.6022 | 0.5066 | 0.1933 | 1.0133 | 0.239 |
| 39 | rs3043804 | 0.4831 | 0.4999 | 0.3747 | 0.386 | 0.614 | 0.5197 | 0.2053 | 1.041 | 0.382 |
| 40 | rs10533337 | 0.5535 | 0.4947 | 0.3721 | 0.4081 | 0.5919 | 0.5478 | 0.2329 | 1.1058 | 0.015 |
| 42 | rs5822909 | 0.4662 | 0.4982 | 0.3739 | 0.3856 | 0.6144 | 0.5159 | 0.2018 | 1.0329 | 0.471 |
| 43 | rs144537609 | 0.5891 | 0.4846 | 0.3669 | 0.3904 | 0.6096 | 0.4991 | 0.1867 | 0.9981 | 0.522 |
| 45 | rs3830564 | 0.454 | 0.4962 | 0.3729 | 0.3667 | 0.6333 | 0.4728 | 0.1648 | 0.9484 | 0.299 |
| 48 | rs67941259 | 0.5216 | 0.4995 | 0.3745 | 0.3736 | 0.6264 | 0.4953 | 0.1834 | 0.9907 | 0.865 |
| 49 | rs79287422 | 0.4719 | 0.4989 | 0.3742 | 0.3752 | 0.6248 | 0.4972 | 0.185 | 0.9944 | 1 |
| 50 | rs142281120 | 0.4756 | 0.4993 | 0.3744 | 0.3767 | 0.6233 | 0.5009 | 0.1883 | 1.0019 | 1 |
| 52 | rs10540867 | 0.4634 | 0.4978 | 0.3737 | 0.3652 | 0.6348 | 0.4728 | 0.1648 | 0.9484 | 0.244 |
| 53 | rs3993057 | 0.5019 | 0.5005 | 0.375 | 0.3854 | 0.6146 | 0.5197 | 0.2053 | 1.041 | 0.395 |
| 57 | rs140025863 | 0.485 | 0.5 | 0.3748 | 0.3741 | 0.6259 | 0.4972 | 0.185 | 0.9944 | 0.932 |
| 58 | rs142623177 | 0.4522 | 0.4959 | 0.3727 | 0.364 | 0.636 | 0.4653 | 0.1589 | 0.9351 | 0.164 |
| 59 | rs142392113 | 0.4878 | 0.5002 | 0.3749 | 0.3786 | 0.6214 | 0.5066 | 0.1933 | 1.0133 | 0.797 |
| 62 | rs55714089 | 0.3799 | 0.4716 | 0.3602 | 0.3906 | 0.6094 | 0.4709 | 0.1633 | 0.945 | 1 |
| 63 | rs35974596 | 0.546 | 0.4962 | 0.3729 | 0.3734 | 0.6266 | 0.4878 | 0.1771 | 0.9762 | 0.713 |
| 64 | rs6144473 | 0.3949 | 0.4784 | 0.3637 | 0.3793 | 0.6207 | 0.4597 | 0.1546 | 0.9253 | 0.379 |
| 65 | rs10544053 | 0.4156 | 0.4862 | 0.3678 | 0.4062 | 0.5938 | 0.531 | 0.2161 | 1.066 | 0.047 |
| 66 | rs10589141 | 0.5009 | 0.5005 | 0.375 | 0.3783 | 0.6217 | 0.5066 | 0.1933 | 1.0133 | 0.784 |
| 67 | rs5821525 | 0.576 | 0.4889 | 0.3692 | 0.3899 | 0.6101 | 0.5066 | 0.1933 | 1.0133 | 0.425 |
| 69 | rs5882232 | 0.4503 | 0.4955 | 0.3725 | 0.3675 | 0.6325 | 0.4728 | 0.1648 | 0.9484 | 0.322 |
| 70 | rs16646 | 0.4578 | 0.4969 | 0.3732 | 0.3677 | 0.6323 | 0.4765 | 0.1678 | 0.9552 | 0.379 |
| 71 | rs5892949 | 0.5216 | 0.4995 | 0.3745 | 0.3736 | 0.6264 | 0.4953 | 0.1834 | 0.9907 | 0.874 |

NOTE: No, the number above the typing profile of the 43 InDel loci; He, expected heterozygosity; PIC, polymorphism information content; MP, match probability; PD, power of discrimination; Ho, observed heterozygosity; PE, power of exclusion; TPI, typical paternity index; *p*HW: *P*-values for the exact tests of Hardy-Weinberg equilibrium.
